# Pancreatic Ascites Complicated by Salmonella Enteritidis Peritonitis: A Diagnostic Challenge and the Role of Surgical Source Control

**DOI:** 10.7759/cureus.116227

**Published:** 2026-09-14

**Authors:** Moussa Sylla, Samuel Bamogo, Eric Pappalardo

**Affiliations:** 1 Surgery, Centre Hospitalier Victor Jousselin, Dreux, FRA

**Keywords:** bacterial peritonitis, case report, exploratory laparoscopy, pancreatic ascites, pancreatic duct disruption, salmonella enteritidis, septic shock

## Abstract

Pancreatic ascites is an uncommon complication of pancreatic disorders resulting from persistent leakage of pancreatic juice into the peritoneal cavity, most commonly due to disruption of the main pancreatic duct or rupture of a pancreatic fluid collection. Bacterial peritonitis complicating pancreatic ascites is rarely reported.

We report the case of a 40-year-old man who presented with massive pancreatic ascites associated with a marked elevation of serum lipase. Analysis of the ascitic fluid strongly supported its pancreatic origin, and conservative management with enteral nutrition and a somatostatin analogue was initiated. One week after biliopancreatic magnetic resonance imaging, the patient developed sudden deterioration with septic shock, tense ascites, acute kidney injury, and purulent ascitic fluid. Emergency laparoscopy was performed because of concern for secondary peritonitis from an occult gastrointestinal perforation. Approximately 9 L of purulent ascites were evacuated, followed by extensive peritoneal lavage and drainage. No gastrointestinal perforation or other intra-abdominal source of contamination was identified. Peritoneal fluid cultures isolated a fully susceptible *Salmonella enterica* serovar Enteritidis, while blood cultures remained negative. Antibiotic therapy was subsequently de-escalated to amoxicillin-clavulanic acid based on antimicrobial susceptibility testing, and a full seven-day course was completed. The postoperative course was favorable. Six weeks later, MRI showed complete resolution of the ascites and a 5-cm organized pancreatic collection communicating with the distal main pancreatic duct.

This case highlights an unusual occurrence of *Salmonella* Enteritidis peritonitis complicating pancreatic ascites in the absence of gastrointestinal perforation or documented bacteremia. Sudden clinical deterioration in patients with pancreatic ascites should prompt immediate reassessment for infectious complications and, when indicated, urgent surgical exploration for source control. Definitive management of the pancreatic ductal leak may be deferred in selected patients when the infectious complication has been controlled and clinical evolution is favorable.

## Introduction

Pancreatic ascites is an uncommon complication of pancreatic disorders resulting from persistent leakage of pancreatic juice into the peritoneal cavity, most commonly due to disruption of the main pancreatic duct or rupture of a pancreatic fluid collection. It is most frequently associated with chronic pancreatitis but may also occur in the setting of acute pancreatitis, pancreatic trauma, or rupture of a pancreatic pseudocyst [1,2].

The diagnosis is based on a combination of clinical, biochemical, and radiological findings. Analysis of ascitic fluid typically demonstrates a protein-rich exudate with a low serum-ascites albumin gradient and markedly elevated pancreatic enzyme levels, particularly amylase. Cross-sectional imaging, including computed tomography and magnetic resonance cholangiopancreatography, is useful for identifying pancreatic duct abnormalities, associated pancreatic collections, and possible sites of ductal disruption [1,2].

Management depends on the patient’s clinical condition and the underlying ductal anatomy. Conservative treatment with nutritional support, pancreatic rest, and somatostatin analogues may be attempted in selected clinically stable patients, although failure rates remain substantial [1]. Endoscopic therapy has an important role in persistent ductal leakage. Transpapillary pancreatic duct stenting is particularly useful when the site of ductal disruption can be bridged, whereas long-term transmural stenting may be considered in selected patients with disconnected pancreatic duct syndrome or associated pancreatic collections [1,3,4]. Surgery is generally considered when endoscopic treatment is inappropriate or unsuccessful, or when complications require urgent source control [1,3].

Bacterial infection complicating pancreatic ascites is rarely reported. Non-typhoidal *Salmonella* can rarely cause peritoneal infection, including in patients without immunosuppression, although previously reported cases have often involved underlying cirrhosis or an identifiable gastrointestinal source [5-7]. In this report, we present a unique diagnostic puzzle: a patient with massive pancreatic ascites who abruptly developed septic shock due to* Salmonella* Enteritidis peritonitis. Strikingly, this severe infection occurred in the complete absence of macroscopic gastrointestinal perforation or documented bacteremia. This case challenges the standard paradigms of both pancreatic ascites management and the typical pathophysiology of bacterial peritonitis, highlighting the vital role of emergency diagnostic laparoscopy. 

## Case presentation

A 40-year-old man undergoing evaluation for hypertension presented to the emergency department with a one-week history of diffuse abdominal pain associated with progressive abdominal distension. One month earlier, he had developed a left lower-limb deep vein thrombosis in the setting of ankle cellulitis and had been treated with apixaban. He had no other relevant medical history.

Initial laboratory testing revealed an inflammatory syndrome and a serum lipase level approximately 10 times the upper limit of normal (Table 1). Abdominal computed tomography (CT) showed massive ascites associated with two pancreatic cystic lesions (Figure 1). Diagnostic paracentesis yielded an exudative fluid with markedly elevated pancreatic enzyme levels, supporting the diagnosis of pancreatic ascites related to a presumed pancreatic ductal leak (Table 1).

**Table 1:** Serial trends of serum and ascitic fluid parameters during the clinical course. (ND: Not Done).

Parameter (Units)	Reference range	Admission	Septic deterioration	Discharge
Serum parameters				
White blood cells (/mm³)	4,000 – 10,000	8,300	38,900	9,000
C-reactive protein (mg/L)	< 5	19	429	5
Creatinine (µmol/L)	60 – 110	66	214	56
Lipase (U/L)	< 60	730	1,200	ND
Albumin (g/L)	35 – 50	ND	29	ND
Ascitic fluid parameters				
White blood cells (/mm³)	-	130	13,400	ND
Total protein (g/L)	-	34	38	ND
Amylase (U/L)	-	1,100	1,300	ND
Lipase (U/L)	-	1,800	>3000	ND
Albumin (g/L)	-	ND	ND	ND

**Figure 1:**
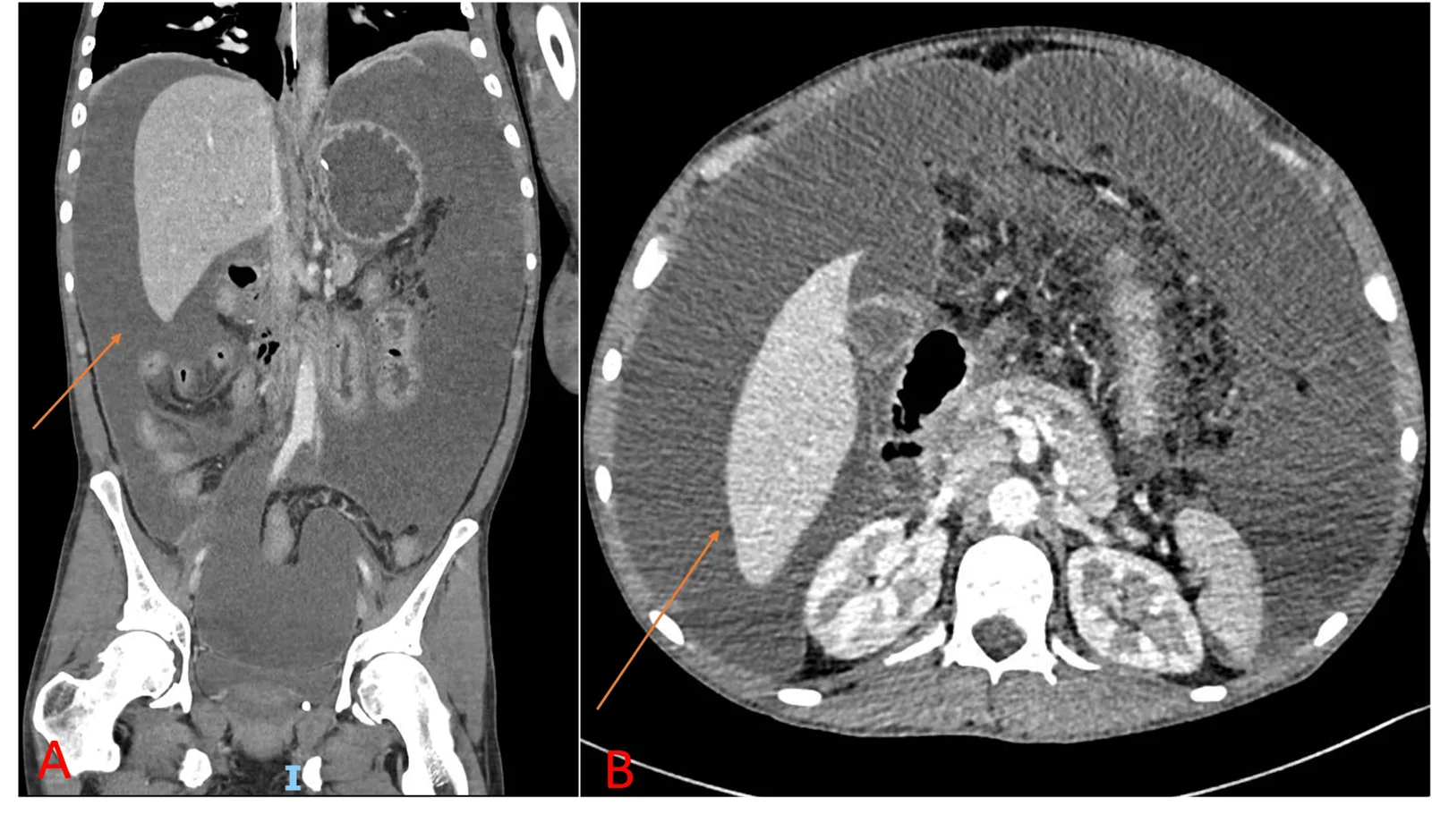
Abdominal computed tomography (CT) images (A: coronal slice, B: axial slice) demonstrating massive ascites (orange arrows)

Following multidisciplinary discussion, conservative treatment was initiated with enteral nutrition administered through a nasogastric tube and continuous intravenous somatostatin infusion, subsequently transitioned to prolonged-release lanreotide.

Biliopancreatic magnetic resonance imaging (MRI) performed one week after admission confirmed abundant free ascites without evidence of chronic liver disease or portal hypertension. It also demonstrated a cephalic cystic lesion suggestive of a branch-duct intraductal papillary mucinous neoplasm and subtle focal cystic dilatation of the pancreatic duct in the body-tail region, without an identifiable obstructive lesion or definite site of ductal disruption (Figure 2).

**Figure 2:**
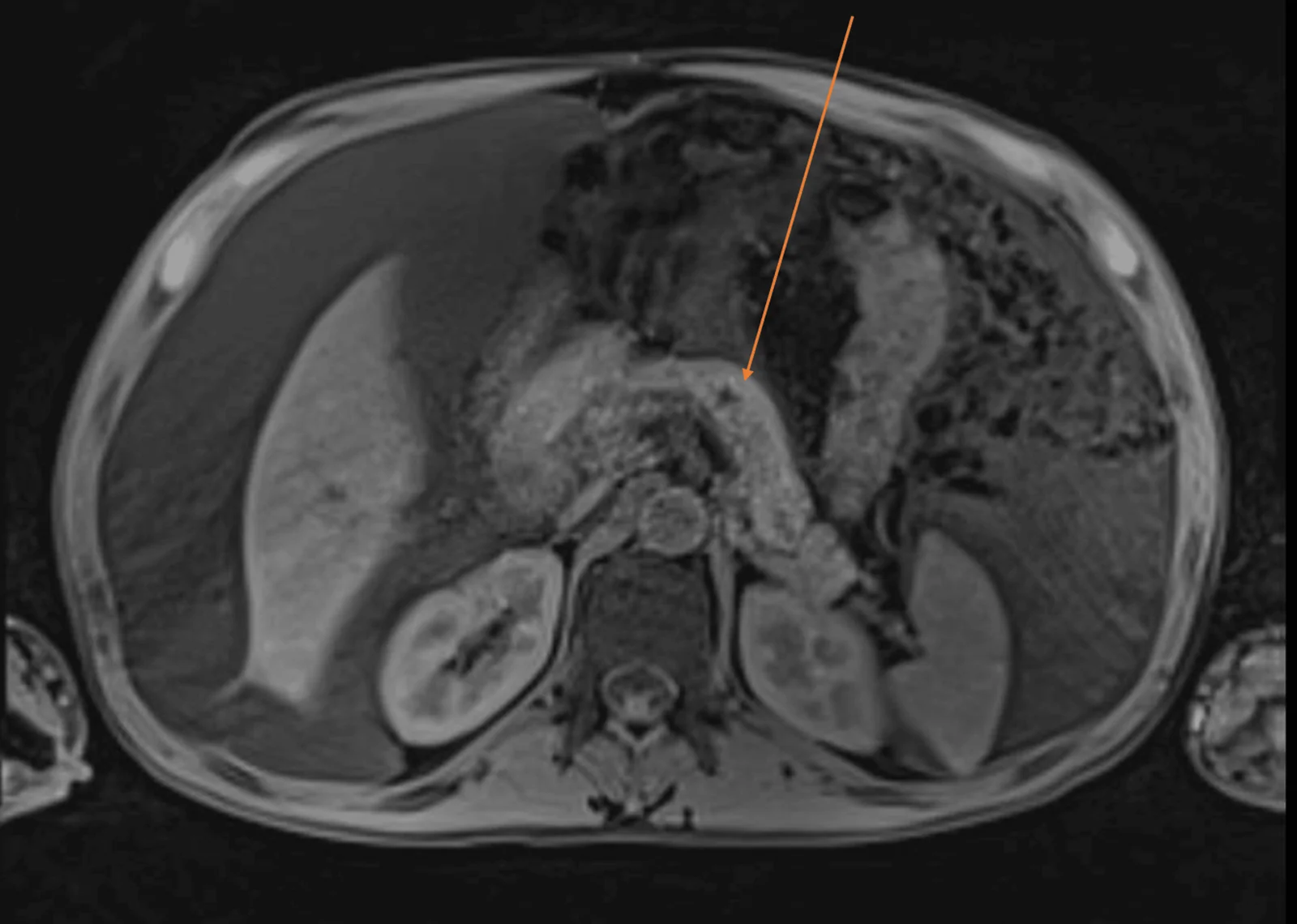
Initial magnetic resonance imaging (MRI) revealing a focal cystic dilation of the pancreatic duct in the body-tail region (orange arrow).

One week after the MRI, the patient developed sudden clinical deterioration characterized by severe abdominal pain, vomiting, tense ascites, hemodynamic instability, and acute kidney injury, consistent with septic shock. Repeat paracentesis yielded purulent fluid with a predominance of polymorphonuclear neutrophils and markedly increased pancreatic enzyme levels (Table 1).

After initial resuscitation, empiric broad-spectrum antibiotic therapy with piperacillin-tazobactam and amikacin was initiated. Given the septic shock and concern for secondary peritonitis from an occult gastrointestinal perforation, emergency laparoscopic exploration was performed. Approximately 9 L of purulent ascites were evacuated, followed by extensive peritoneal lavage and drainage. No gastrointestinal perforation or other intra-abdominal source of contamination was identified.

Peritoneal fluid cultures subsequently isolated *Salmonella enterica *serovar Enteritidis. Antimicrobial susceptibility testing demonstrated a fully susceptible isolate. Blood cultures remained negative. In the absence of gastrointestinal perforation, another identifiable intra-abdominal source, or documented bacteremia, the findings were considered consistent with *Salmonella* Enteritidis peritonitis complicating pancreatic ascites.

On postoperative day 3, based on antimicrobial susceptibility testing, antibiotic therapy was de-escalated to amoxicillin-clavulanic acid to complete a total seven-day course. The postoperative course was favorable, with resolution of septic shock, normalization of renal function, and regression of the inflammatory syndrome within one week (Table 1).

Follow-up MRI performed six weeks later showed complete resolution of the ascites and the development of a 5-cm organized pancreatic collection in contact with the pancreatic tail and communicating with the distal segment of the main pancreatic duct (Figure 3). At follow-up, the patient was asymptomatic, with no recurrence of ascites. Enteral nutrition and somatostatin analogue therapy were discontinued, and clinical and radiological surveillance of the residual pancreatic collection was chosen.

**Figure 3:**
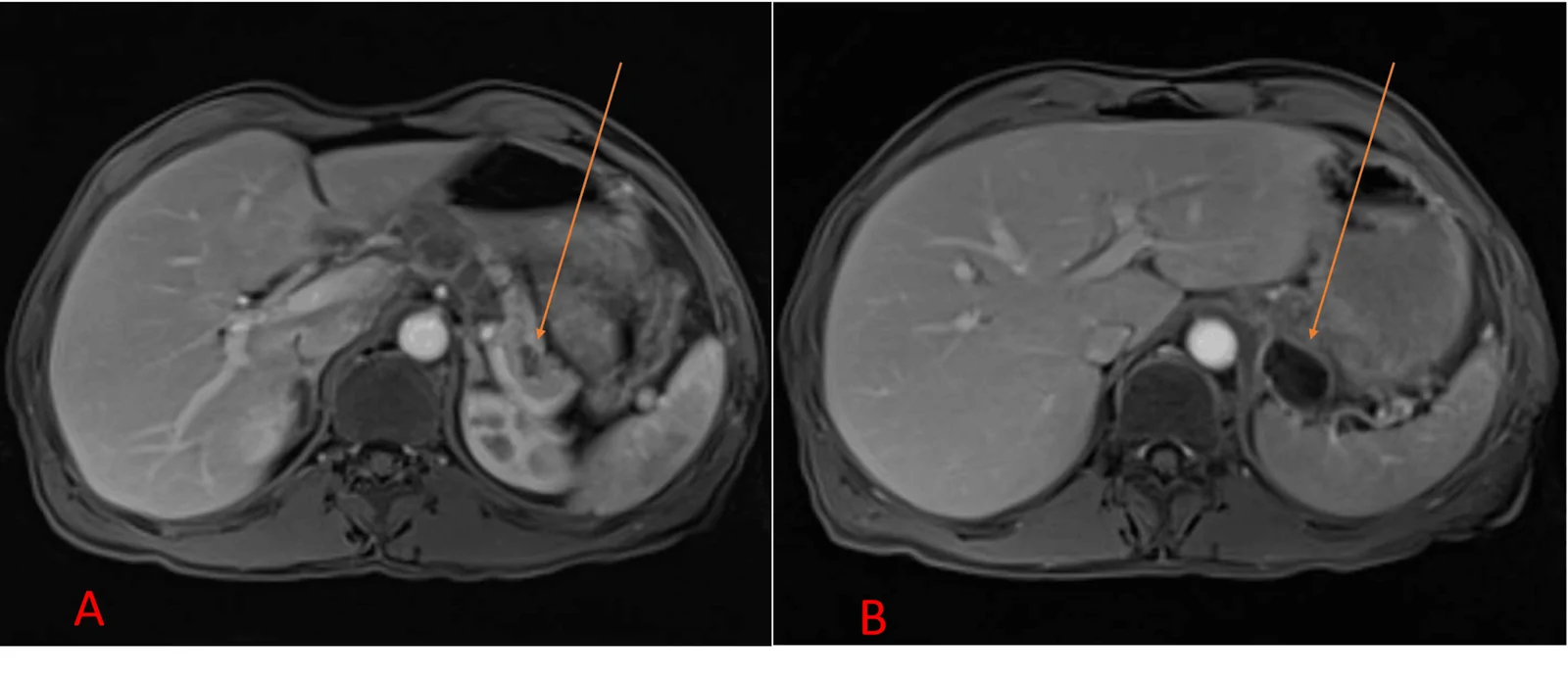
Follow-up magnetic resonance imaging (MRI) revealing a focal cystic dilation of the pancreatic duct in panel A (orange arrow) and a 5-cm organized pancreatic collection in contact with the tail of the pancreas in panel B (orange arrow).

## Discussion

Pancreatic ascites is an uncommon complication of pancreatic ductal disruption characterized by persistent leakage of pancreatic fluid into the peritoneal cavity. It is most frequently associated with chronic pancreatitis but may also occur in the setting of acute pancreatitis, pancreatic trauma, or rupture of a pancreatic fluid collection [1,2]. Diagnosis is based on the clinical presentation, biochemical analysis of ascitic fluid, and pancreatic imaging. Markedly elevated pancreatic enzyme levels in ascitic fluid, particularly amylase, together with compatible imaging findings, strongly support the diagnosis [1,2].

In the present case, the initial diagnosis was challenging because the patient had no known history of chronic pancreatitis and presented with massive ascites associated with pancreatic cystic abnormalities. The markedly elevated pancreatic enzyme concentration in the ascitic fluid supported a pancreatic origin. Initial magnetic resonance imaging demonstrated focal cystic dilatation of the pancreatic duct in the body-tail region but did not identify a definite site of ductal disruption. Six weeks later, follow-up imaging demonstrated an organized pancreatic collection communicating with the distal main pancreatic duct. This subsequent finding provided additional anatomical support for pancreatic ductal disruption as the underlying mechanism of the ascites.

The management of pancreatic ascites remains heterogeneous because of the rarity of the condition and the absence of high-quality comparative trials [1]. Conservative treatment may be attempted in clinically stable patients and generally includes nutritional support, reduction of pancreatic stimulation, therapeutic paracentesis when required, and, in selected cases, somatostatin analogues [1,2]. However, conservative management has been associated with substantial failure rates, particularly when a persistent ductal disruption is present [1]. Endoscopic treatment is therefore an important therapeutic option when the ductal anatomy is suitable. Transpapillary pancreatic duct stenting aims to decompress the pancreatic duct and facilitate closure of the leak, particularly when the stent can bridge the site of disruption [1,3]. In a series of eight patients with pancreatic ascites, transpapillary stenting resulted in resolution of ascites in seven patients within six weeks [3]. More recent consensus recommendations support endoscopic treatment in appropriately selected patients, particularly when the ductal disruption can be traversed endoscopically, while long-term transmural stenting may be considered in selected patients with disconnected pancreatic duct syndrome [4]. Surgery remains an option when endoscopic therapy is not feasible or unsuccessful, or when the underlying ductal anatomy requires definitive surgical treatment [1,4].

In our patient, the initially stable clinical condition and the absence of a clearly identifiable site of ductal disruption led to a conservative strategy following multidisciplinary discussion. The subsequent sudden clinical deterioration markedly changed the therapeutic priorities. Severe abdominal pain, vomiting, tense ascites, hemodynamic instability, acute kidney injury, and purulent ascitic fluid with marked neutrophilic inflammation were highly suggestive of a severe intra-abdominal infectious complication. Because an occult gastrointestinal perforation could not initially be excluded, emergency laparoscopic exploration was performed.

The most unusual feature of this case was the isolation of *Salmonella enterica* serovar Enteritidis from the purulent ascitic fluid. Peritoneal infection caused by non-typhoidal *Salmonella* is uncommon and has been described particularly in patients with cirrhosis or other predisposing conditions [5,6]. However, *Salmonella* Enteritidis peritonitis has also been reported in an immunocompetent patient, although the previously reported case was associated with spontaneous intestinal perforation [5]. In another report involving cirrhotic patients, *Salmonella *Enteritidis was isolated from ascitic fluid without clinical or microbiological evidence of *Salmonella* infection elsewhere [6]. A further case of *Salmonella* Enteritidis bacterascites was reported in a patient with cirrhosis and diabetes, with negative blood cultures, although the clinical context was substantially different from that of our patient [7].

Our case differs from these reports in several respects. The patient had no cirrhosis, immunosuppression, diabetes, or other relevant comorbidity. He had no preceding diarrheal illness, and no stool culture was performed because there was no clinical indication. Only two paracenteses were performed. Most importantly, emergency laparoscopy found no gastrointestinal perforation or other intra-abdominal source of contamination, while blood cultures remained negative. The combination of microbiologically documented *Salmonella* Enteritidis peritonitis, absence of an identifiable gastrointestinal source, and negative blood cultures therefore represents an unusual clinical presentation.

The precise mechanism by which *Salmonella* Enteritidis reached the pancreatic ascitic fluid remains uncertain. Bacterial translocation from the gastrointestinal tract is a possible mechanism described in *Salmonella* peritoneal infections, particularly in patients with underlying conditions that impair intestinal barrier integrity or host defense [7]. However, the absence of diarrhea or another documented gastrointestinal infection in our patient provides no evidence for a specific enteric source. Procedure-related inoculation following paracentesis can also be considered as a theoretical possibility, but the limited number of procedures and the absence of microbiological evidence linking the procedures to the infection do not establish a causal relationship. Accordingly, the route of infection remains speculative.

The surgical intervention was primarily aimed at urgent infectious source control rather than definitive treatment of the pancreatic ductal disruption. Laparoscopy allowed evacuation of approximately 9 L of purulent ascites, extensive peritoneal lavage, and drainage, while simultaneously excluding gastrointestinal perforation. No major pancreatic resection was undertaken during the acute septic episode. This approach avoided a potentially morbid pancreatic procedure in the setting of severe systemic inflammation and septic shock. More broadly, contemporary management of severe pancreatic complications favors minimally invasive and step-up approaches over immediate open pancreatic surgery when intervention is required, particularly in the setting of infected pancreatic collections [8,9].

The subsequent clinical course supported the decision to defer definitive pancreatic intervention. Six weeks after the acute episode, follow-up magnetic resonance imaging demonstrated complete resolution of the ascites and a 5-cm organized pancreatic collection communicating with the distal main pancreatic duct. Because the patient was asymptomatic and had no recurrent ascites, enteral nutritional support and somatostatin analogue therapy were discontinued, and clinical and radiological surveillance was chosen. The management of organized pancreatic collections is generally guided by symptoms, infection, complications, anatomy, and clinical evolution rather than by size alone [8,9]. In our patient, the absence of persistent symptoms or recurrent ascites supported continued observation rather than immediate endoscopic or surgical intervention.

The main limitation of this case is that the precise route of *Salmonella* Enteritidis infection could not be established. No gastrointestinal perforation was identified, blood cultures were negative, and there was no clinical history of diarrheal illness. In addition, the precise site of pancreatic duct disruption was not demonstrated during the initial evaluation and became apparent only on subsequent imaging. Nevertheless, the combination of purulent pancreatic ascites, positive culture for *Salmonella* Enteritidis, negative blood cultures, exclusion of gastrointestinal perforation at surgery, and subsequent demonstration of communication between the pancreatic collection and the main pancreatic duct makes this observation clinically unusual.

This case emphasizes that sudden septic deterioration in a patient with pancreatic ascites should prompt immediate reassessment for bacterial peritonitis and other intra-abdominal sources requiring urgent source control. In our patient, emergency surgical exploration provided both source control and exclusion of gastrointestinal perforation, while avoiding major pancreatic surgery during septic shock. The subsequent favorable clinical and radiological evolution allowed definitive treatment of the pancreatic ductal abnormality to be deferred. This case therefore illustrates that management should be individualized according to the patient's clinical status, the presence or absence of infection, and the anatomical characteristics and subsequent evolution of the pancreatic ductal disruption.

## Conclusions

Pancreatic ascites is an uncommon complication of pancreatic ductal disruption that may be complicated by severe intra-abdominal infection. In this case, the development of septic shock was associated with purulent pancreatic ascites and isolation of *Salmonella enterica* serovar Enteritidis, despite negative blood cultures and the absence of gastrointestinal perforation or another identifiable intra-abdominal source. The mechanism responsible for the infection remains uncertain. Although bacterial translocation and procedure-related inoculation following paracentesis are possible hypotheses, the available data do not establish a causal mechanism.

This case emphasizes that sudden clinical deterioration in a patient with pancreatic ascites should prompt rapid reassessment for an infectious complication. In the setting of septic shock, early resuscitation, appropriate antimicrobial therapy, and urgent surgical exploration when a surgically treatable source cannot be excluded are essential for adequate source control. In our patient, laparoscopic evacuation and drainage controlled the infectious complication without requiring major pancreatic surgery, allowing subsequent conservative management of the pancreatic ductal abnormality.
